# Safety evaluation of the interchangeable use of robenacoxib (Onsior™) tablets and solution for injection in dogs

**DOI:** 10.1186/s12917-017-1269-z

**Published:** 2017-11-28

**Authors:** Céline E. Toutain, Mark C. Heit, Stephen B. King, Rainer Helbig

**Affiliations:** 1 Elanco Animal Health, a Division of Eli Lilly and Company, Mattenstrasse 24A, CH-4058 Basel, Switzerland; 2 Elanco Animal Health, a Division of Eli Lilly and Company, 2500 Innovation Way, Greenfield, IN 64140 USA

**Keywords:** Robenacoxib, Onsior™, Tablet, Injection, NSAID, Dog, Safety, Interchangeable

## Abstract

**Background:**

Robenacoxib (Onsior™) is a non-steroidal anti-inflammatory drug developed for canine and feline use for the control of pain and inflammation. It is available as both tablets and solution for injection. The objective of this safety study was to investigate the interchangeable use of two robenacoxib formulations in dogs using a novel study design alternating between oral tablets and subcutaneous injections. Thirty-two naïve healthy 4-month dogs were enrolled in this 88-day study and were randomized among four groups to be untreated or to receive robenacoxib at the highest recommended or elevated dose rates. The dogs were administered three 20-day treatment cycles each separated by a 14-day washout period. Each 20-day cycle was comprised of 10 days of once daily oral administration, 3 days of subcutaneous administration, followed by further 7 days of oral administration (Groups 2 to 4). The control group (Group 1) received oral empty gelatin capsules or subcutaneous saline injections. Assessment of safety was based on general health observations, clinical observations, physical and neurological examinations including ophthalmological examinations, electrocardiographic examinations and clinical pathology evaluations, food and water consumption, body weight, and macroscopic and microscopic examinations. Blood samples were collected for pharmacokinetic evaluation.

**Results:**

Blood concentrations of robenacoxib confirmed systemic exposure of all treated dogs. All dogs were in good health through study termination and there were no serious adverse events during the course of the study. No changes in body weight, food consumption, ophthalmic, neurological examinations, electrocardiograms, buccal mucosal blood times, clinical pathology or organ weight were attributable to robenacoxib formulation administration. Primary treatment-related abnormalities were of low incidence at all doses. They were confined to macroscopic and microscopic changes observed locally at the subcutaneous injection sites and microscopic findings within the gastrointestinal tract. These findings were as expected based on previous studies with robenacoxib solution for injection alone and the known properties of this class of compound and mode of administration. There were no adverse effects which could be attributed specifically to the interchangeable use of oral and injectable robenacoxib.

**Conclusions:**

Alternating regimens of robenacoxib tablets and solution for injection were well tolerated in healthy young dogs.

## Background

Robenacoxib is a non-steroidal anti-inflammatory drug (NSAID) of the cyclooxygenase (COX)-2 inhibitor (coxib) subclass with a fast onset of action in dogs and cats [1, 2]. It is a registered veterinary medicinal product available for dogs as an injectable (Onsior™ 20 mg/mL solution for injection, Elanco Animal Health, Greenfield, IN) and oral (Onsior™ 10 mg, 20 mg and 40 mg tablets, Elanco Animal Health, Greenfield, IN) formulation. Onsior™ is licensed in the US for use in dogs for the control of postoperative pain and inflammation associated with soft tissue surgery at a dose of 2 mg/kg subcutaneously or 2 mg/kg orally (2–4 mg/kg range) for a maximum of 3 days [3, 4]. Onsior™ tablet is registered in Europe for the treatment of pain and inflammation associated with chronic osteoarthritis in dogs at a dose of 1 mg/kg (1–2 mg/kg range) for up to 10 days. Onsior™ is also available in Europe as a solution for injection for the treatment of pain and inflammation associated with orthopedic or soft tissue surgery at a dose of 2 mg/kg [5]. The systemic bioavailability of robenacoxib solution is 88% and it is 62% with food or 84% without food, for the tablet [5].

Thanks to its pharmacodynamic and pharmacokinetic properties, robenacoxib displays a favorable safety profile in healthy dogs [6]. Indeed, robenacoxib targets COX-2 activity and is able to spare COX-1 activity [1, 6–8]. In addition, robenacoxib is characterized by a rapid elimination from the central compartment with a prolonged persistence at sites of inflammation [9, 10].

The safety of robenacoxib has been investigated in both cats [11, 12] and dogs [6, 13]. The safety of oral administration was demonstrated in individual studies in healthy young dogs (approximately 6 months of age) up to 40 mg/kg for 1 month and up to 10 mg/kg for 6 months [6]. In addition, the safety of subcutaneous administration in adult dogs was established up to 10 mg/kg once a day for 3 days [4].

This target animal safety study was a component of the safety data package presented to the Food and Drug Administration’s Center for Veterinary Medicine (FDA-CVM) for the interchangeable use approval. Its objective was to apply a novel study design to establish the safety of the interchangeable use of Onsior™ tablets and Onsior™ solution for injection in dogs. By alternating between oral tablet and subcutaneous (SC) injection dosing, it was possible to demonstrate that tablets and injections can be used sequentially in either order. This is relevant for situations, in which for example a dog receives robenacoxib for surgery via injection and then continues for two additional days of postoperative pain therapy with tablets or when a dog treated with oral robenacoxib for osteoarthritis receives a single injection of robenacoxib for surgery and then continues with its oral osteoarthritis therapy. Because Onsior™ is a globally marketed product and dosages and indications vary in different geographies, the dose levels used in this study were selected to equal or exceed all potential scenarios of use and addressed both indications (postoperative pain and osteoarthritis). Group 1 was the negative control group. Groups 2 to 4 received three cycles of oral and subcutaneous treatments (separated by a washout period). During each cycle, Group 2 was administered ≥2 mg/kg orally for 7 days, ≥4 mg/kg orally for 3 days, 4 mg/kg subcutaneously for 3 days and ≥2 mg/kg orally for 7 days. Groups 3 and 4 received multiples thereof (2X and 3X). In addition, to investigate the minimum proposed label age for safe use of Onsior™, dogs were 4 months of age at start of treatment compared to 6 months of age in previous safety studies.

## Methods

### Objective and standards

The objective of the study was to establish the safety of the interchangeable use of robenacoxib (Onsior™) tablets and solution for injection in dogs starting at 4 months of age.

This study was conducted in compliance with Good Laboratory Practice [14, 15] and site procedures. It was designed in consultation with regulatory authorities (FDA-CVM) and according to the VICH guideline for evaluating the target animal safety of new pharmaceuticals (VICH Guideline 43 [16]). All animal procedures were in compliance with the U.S. Department of Agriculture’s Animal Welfare Act [17], the Guide for the Care and Use of Laboratory Animals [18] and the Office of Laboratory Animal Welfare [19]. The study was reviewed and approved by the study site Institutional Animal Care and Use Committee.

### Animal management

Young purpose-bred mongrel dogs (crossbred hounds) were used. Thirty-two naïve dogs (16 males and 16 females, weighing 13 kg to 19.3 kg and 11.9 kg to 13.8 kg, respectively at initiation of dosing) were selected and obtained commercially from a laboratory animal supplier (Marshall BioResources, North Rose, New York). Each animal was uniquely identified with a microchip and acclimatized to the study conditions for 4 weeks. Dogs were approximately 4 months old at initiation of dosing (Day 1). Suitability was evaluated by physical examination and clinical pathology and only healthy animals were included. Dogs were housed individually indoors in climate controlled facilities (20 to 26 °C, 30 to 70% humidity and 12-h light/12-h dark cycle) in accordance with accepted laboratory animal care and use guidelines. Dogs were fed ad libitum at least 8 h each day with an appropriate ration of a certified commercial canine feed and water was available ad libitum. Dogs were observed for general health, behavior and appetite at least once daily throughout the study. Environment enrichment included visual, auditory and olfactory contact with other animals and dogs were provided exercise opportunities in large kennel runs.

### Experimental design

**Fig. 1:**
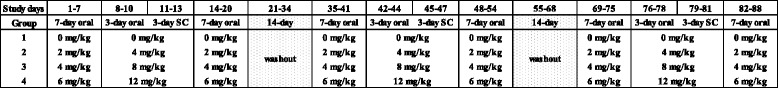
Study design diagram

This was a randomized, controlled, blinded study with a parallel design (Fig. 1). The 32 dogs were randomized to the four dose groups and the four rooms used in the study within each sex, with an equal number of males and females within each room and an equal number of animals from each group within each room. Healthy mongrel dogs (4/sex/group) were administered three 20-day cycles (each separated by a 14-day washout period). Each 20-day cycle was comprised of 10 days of once daily oral robenacoxib administration, 3 days of subcutaneous robenacoxib treatment followed by 7 days of oral administration (Groups 2 to 4) to result in a total of 60 treatment days and 88 in-life days. The negative control group (Group 1) received empty gelatin capsules or saline injection consistent with the Group 4 animals.

### Test articles and administration

Robenacoxib was orally administered as Onsior™ tablets (20 or 40 mg of robenacoxib) or by subcutaneous administration of Onsior™ solution for injection (20 mg/mL of robenacoxib) under fasted conditions. The excipients used in the Onsior™ formulations [5] are standard excipients and are widely used in veterinary and human pharmaceuticals. Appropriately sized whole tablets placed in one or two gelatin capsules (Torpac® no.12, Torpac Inc., Fairfield, NJ, USA) were used to provide the most accurate dosing based on the body weights of the study dogs. The number and strength of oral tablets were calculated to ensure that the minimum targeted dose level was achieved. For Group 2, this meant that dogs received a robenacoxib dose of at least 2 mg/kg during the first and last 7 days of each cycle and at least 4 mg/kg from the 8th to 13th day of each cycle (Fig. 1). The dose level for robenacoxib injections was in all cases delivered at the exact dosage (i.e. 4 mg/kg for Group 2). Dose levels within the study were determined as multiples (2X and 3X) of the dose levels received by Group 2. A different injection site was used on each dosing day (as recommended by the Joint Working Group on Refinement [20] and the product label [4]) in order to minimize the potential damage that could result from multiple injections at a single site. Injections were administered at nine distinct sites where three each were located at the left shoulder, the midline interscapular region and right shoulder.

### Safety variables

Safety variables recorded and samples collected during the in-life phase are presented in Table 1. Clinical observations, body weight, food consumption, physical and neurological examinations, injection site scoring, ophthalmologic examinations, electrocardiographic examinations, buccal mucosal bleeding time (BMBT) and clinical pathology evaluations (hematology, clinical chemistry, coagulation and urinalysis) were performed prior to randomization, during and at the end of the dosing phase.

**Table 1:** Safety variables recorded and samples collected during the in-life phase

Variables	Study days^a^
Clinical observations	General observations twice daily Cageside observations once daily Observations 1 h ± 15 min postdose Detailed observations (Days 7, 10, 13, 21, 28, 34, 41, 44, 47, 55, 62, 69, 75, 78, 81 and 89) 1 h ± 15 min postdose (on dosing days)
Body weight	Days 1, 8, 11, 14, 21, 28, 35, 42, 45, 48, 55, 62, 69, 76, 79, 82, 88 and 89
Food consumption	Daily
Physical and neurological examinations	Days 20, 34, 54, 68 and 88
Injection sites scoring	Days of injection (predose and 7 to 8 h postdose) and at 24, 48, and 72 h (±1 h) following each injection
Ophthalmologic examination	Day 88
Electrocardiographic examination	1 h ± 15 min postdose on Days 20 and 88
Buccal mucosal bleed times	Days 20 and 88
Clinical pathology	Days 21, 55 and 89
Bioanalytical collection	*Dose cycle 1:* On days 1 and 14, samples were collected predose and 1 and 5 h postdose On days 6, 8 and 11, samples were collected 1 and 5 h postdose *Dose cycle 3:* On days 69 and 82, samples were collected predose and 1 and 5 h postdose On days 74, 76 and 79, samples were collected 1 and 5 h postdose

^a^Dosing phase length was 88 days and necropsies were performed at study termination at Day 89

Animals were checked twice daily for mortality, abnormalities and signs of pain or distress. Cageside observations were made once daily throughout the study. Additionally, on each day of dosing, observations were performed 1 h (± 15 min) post-dose and on each day of subcutaneous dosing, animals were observed for an immediate response upon administration of the injection. Detailed observations included, but were not limited to: changes in skin, fur, eyes, and mucous membranes; occurrences of secretions and excretions; and autonomic activity (e.g., lacrimation, piloerection, pupil size and unusual respiratory pattern). For evaluation of gait, animals were allowed to walk freely.

Body weights were measured at least once a week during the study, at the same time in the morning (prior to dosing) on a calibrated balance. Food consumption was measured and recorded daily. Complete physical and neurological examinations were conducted five times during the study, by a veterinarian. Physical examinations included evaluation of general condition and behaviour, eyes without ophthalmoscope, integument, musculoskeletal and gastrointestinal systems, body temperature, cardiovascular and respiratory systems, including calculation of heart rate and assessment by auscultation and reproductive system. Neurological examinations included observation of nystagmus, pupillary response, extensor thrust (muscle tone), righting reflex, startle reflex and walking movement.

Injection sites were scored for erythema, edema, heat and pain on palpation and were measured (if necessary), on days of dosing and for 3 days following each injection. Electrocardiograms (ECGs) were performed to evaluate the cardiovascular system in accordance with VICH GL 43. They were digitally recorded from unanesthetized animals on Days 20 and 88 and the ECG traces from each animal were examined by a certified veterinary cardiologist for the following variables: PR interval, QRS duration, QT interval, RR interval, heart rate and heart rate-corrected QT (QTc) interval. A qualitative review for rhythm abnormalities and disturbances was performed.

### Analyses of robenacoxib concentrations in blood

Whole blood (2 mL) was collected from fasted animals via a jugular or cephalic vein 1 and 5 h post dose on Days 1, 6, 8, 11, 14, 69, 74, 76, 79 and 82 (Table 1). Additionally, pre-dose samples were collected within 30 min prior to dose administration on Days 1, 14, 69 and 82. Samples were placed in tubes containing K_2_ EDTA and were stored frozen (−10 to −30 °C) within 1 h after collection. Whole blood rather than plasma was chosen as smaller volumes of blood are needed which is an advantage not to interfere with primary safety endpoints and in terms of animal welfare. The blood: plasma concentration ratio is approximately 0.5: 1 in the dog, indicating lower concentration in blood than in plasma and that robenacoxib does not penetrate into cells [21] .

Robenacoxib was quantitatively analyzed in blood using a validated analytical method involving liquid chromatography with tandem mass spectrometry detection (LC-MS/MS). Each blood sample (50 μL) was prepared using protein precipitation and chromatographed by HPLC on an ACE 5 μm C18 column (50 × 2.1 mm). The samples were analyzed on an Applied Biosystems/Sciex API 5000 triple quadrupole mass spectrometer system using the negative electrospray ionisation (TurboIonSpray®) mode and multiple reaction monitoring of the transition m/z 326 → 222 for robenacoxib. The method utilized an internal standard (i.e., deuterated robenacoxib) and quantification ranged from 2.00 ng/mL (lower limit of quantitation; LLOQ) to 2000 ng/mL (upper limit of quantitation) based on peak area using linear regression with 1/x^2^ concentration weighting. Performance of the method was monitored by quality control samples at 6, 80 and 1600 ng/mL. Mean inter-day precision was 13.0% at LLOQ and ranged between 6.9 and 9.4% at the other levels and the mean inter-day accuracy ranged between 96.5 and 104.2%.

### Pharmacokinetics and pharmacodynamics of robenacoxib

Only abbreviated pharmacokinetic sampling was performed so as not to interfere with the primary safety objective of the study. The limited sampling schedule did not allow calculation of half-life or extrapolation of AUC for the entire dose period (i.e., 0 to 24 h), therefore, exposure between 1 and 5 h was calculated (AUC_1-5h_) using the loglinear trapezoidal rule. Concentrations below the limit of quantification were considered 0.

For levels of inhibition of COX-1 and COX-2, the standard sigmoid model:

$$ \%\mathrm{Inhibition}=\left({\mathrm{I}}_{\mathrm{max}}\times {\mathrm{C}}^{\mathrm{n}}\right)/\left({\mathrm{C}}^{\mathrm{n}}+\mathrm{IC}{50}^{\mathrm{n}}\right) $$

was used to predict inhibition of TxB_2_ (as an index of COX-1 inhibition) and PGE_2_ (as an index of COX-2 inhibition) at the robenacoxib concentrations observed during the study. In this model, I_max_ is the maximal inhibition (100%), IC50 is the concentration of robenacoxib providing 50% of Imax, C is the robenacoxib concentrations and n is the slope parameter. The parameters values were obtained from previously published work in dog [6, 8]. Mean values for n and IC50 were, respectively, 0.586 and 11,328 ng/mL for inhibition of TxB_2_ and 1.273 and 280.3 ng/mL for inhibition of PGE_2_. Inhibition level means and standard deviations at 1 and 5 h after treatment were calculated for each dose. The percent inhibition versus dose was analysed using analysis of variance and the statistical software GraphPad Prism 7.02 (GraphPad Software, La Jolla California USA).

### Pathological examinations

At study termination on Day 89, complete necropsies were performed on all animals and macroscopic abnormalities for all tissues were recorded. Organs were weighed and tissues were processed for microscopic examination.

### Statistical evaluations of safety variables

All data were analyzed with the statistical software package SAS (2002–2008) (Statistical analysis system. Version 9.2, Cary, North Carolina: SAS Institute Inc.). The following endpoints were analyzed: body weight, food consumption, injection site scoring, ECG variables, organ weights, clinical pathology and pharmacokinetic parameters. Except for the pharmacokinetic parameters, each treated group (Group 2 to 4) was analyzed compared to the control group (Group 1).

Endpoints measured once post-treatment (i.e., organ weight) were analyzed using analysis of variance (ANOVA) with treatment, sex and treatment by sex as fixed effects [22]. If a pre-treatment value was available (i.e., ECG), it was used as a covariate (analysis of covariance, ANCOVA). Endpoints measured multiple times postdose (i.e. injection site scoring) were analyzed using repeated measures analysis of variance (RMANOVA) with treatment, time and sex, the two-way interactions treatment x time, treatment x sex, and sex x time, and the three-way interaction treatment x time x sex all as fixed effects. If a pre-treatment measurement was available (i.e., bodyweights, food consumption, hematology, coagulation, urinalysis and clinical chemistry), it was used as a covariate (repeated measures analysis of covariance, RMANCOVA). The pre-treatment value closest to dosing was used as the covariate [23].

Depending on the significance of the interaction terms (*p* ≤ 0.10 level for two-way interactions and *p* ≤ 0.05 for the three-way interaction), treated groups were compared to the control either within each sex (treatment x sex significant), within each time point (treatment x time significant) or main effect only (neither treatment x sex nor treatment x time significant).

## Results

### Test article dosages

Ranges for individual robenacoxib dosages achieved using tablets are provided in Table 2 and were equal to or exceeded the minimum target oral dose levels in all treatment groups. Due to body weight gain of study dogs, the individual dose volumes of injected sterile saline (0.6 mL/kg) or robenacoxib solution (0.2, 0.4 and 0.6 mL/kg for Group 2 to 4) increased from the first dosing cycle through the last dosing cycle in all animals. Ranges for individual injected dose volumes are provided in Table 3 where the maximum that any dog received was 17.0 mL. The maximum dose volume of 0.6 mL/kg administered in this study was below the administration volume considered good practice or maximal (1 and 2 mL/kg respectively) [24].

**Table 2:** Oral dosing table

Group	Target dosage (mg/kg/day)	Actual dosage range (mg/kg/day)
Group 2	≥2	2.0–3.4
	≥4	4.1–5.3
Group 3	≥4	4.0–5.3
	≥8	8.0–9.1
Group 4	≥6	6.0–7.3
	≥12	12.0–13.4

**Table 3:** Subcutaneous dosing volume table

Group	Study day	Dose volume (mL/day)
Group 1	11–13 45–47 79–81	7.9–10.9 9.8–14.9 10.9–16.7
Group 2	11–13 45–47 79–81	2.6–4.3 3.3–5.6 3.7–6.4
Group 3	11–13 45–47 79–81	5.5–7.7 6.9–10.0 7.6–11.7
Group 4	11–13 45–47 79–81	7.8–11.3 10.4–14.4 11.0–17.0

### Physical, clinical and neurological observations

All mongrels were in good health through study termination. There were no serious adverse events during the course of the study and no treatment-related adverse findings in general health observations. No treatment-related abnormalities were noted during the physical examinations or neurological abnormalities other than injection site findings as described hereafter. Over the entire study, a total of 288 subcutaneous injections were administered and only occasional scratching (47 observations) or excessive licking of the dose site (27 observations) were noted. Additionally, there was a single observation each of twitching and vocalization, in a Group 4 female and Group 2 male, respectively during dosing.

Other clinical observations included liquid, mucoid, or non-formed feces, vomitus and various other findings on the skin and pelage. These observations appeared infrequently, were transient and lacked a dose relationship for incidence, or were reported at a comparable incidence in control animals and/or during the predose phase or washout periods. They were therefore not considered test article-related.

### Body weights and food consumption

All dogs gained weight during the study. Individual body weight gain in the treatment groups between dosing phase Day 1 and 88 ranged from 8.1 to 15.5 kg and 6.4 to 10.8 kg for males and females, respectively. No test article-related effects were noted in body weight for either sex as no statistically significant differences between control and treated animals were observed (*p* = 0.2386). A significant sex x time interaction was observed (*p* = 0.0002), which is expected in growing animals. No test article-related effects were noted in food consumption as no statistical significant differences between control and treated animals were observed (*p* = 0.1233).

### Ophthalmology, electrocardiography and buccal mucosal bleeding time

No ophthalmic or ECG parameter abnormalities or changes in BMBT were noted during the study. During the two pre-test periods, BMBT ranged from 34 s to 2 min 44 s in all study animals. On Day 20 and 88, the times for all animals were within this range with two exceptions. On Day 20, the BMBT for a Group 2 female was 3 min and 16 s, but decreased to 1 min and 26 s on Day 88. This female had increased fibrinogen on Day 21 likely associated with the concurrent findings of a scab and swollen skin at the injection site on Days 19 and 21. On Day 88 the BMBT for a Group 3 female was 25 s which was considered not clinically relevant.

### Injection sites

There was a greater number of injection sites with edema at 8 h post-injection and with erythema at 24, 48, and 72 h post-injection in Groups 3 and 4 compared to Groups 1 or 2. In some instances, the erythema continued as skin thickening and/or an ulcer/cyst developed followed by granulation. Ulcers or fluid-filled cysts occurred in one control dog, 2 dogs in Group 2, 4 dogs in Group 3 and in 4 dogs in Group 4. Ulcers tended to have a delayed occurrence, happening 6–8 days after injection out to 10–14 days after injection.

Microscopic findings at injection sites included subcutaneous necrosis, degeneration, and/or fibrosis with occasional involvement of the underlying panniculus muscle (Group 2–4). Minimal to moderate acanthosis/hyperkeratosis of the overlying skin was observed (Group 2–4). Minimal or slight acanthosis/hyperkeratosis in two Group 3 dogs and one Group 4 dog, and minimal to moderate ulceration of the skin (Groups 3 and 4) were also observed. Slight infiltrates of macrophages and an infiltrate of lipid within the axillary lymph nodes were noted in one control dog and all treated dogs. Axillary lymph node findings reflected drainage from the subcutaneous injection sites, therefore, they were considered secondary and not primary test article-related effects.

### Clinical pathology and urinalysis

Summary data for selected haematology and clinical chemistry are presented in Tables 4 and 5. There were no notable differences present for haematology, clinical chemistry, coagulation profiles, or urinalysis parameters between control and treated groups. Any statistically significant differences (e.g. lower white blood cell count for Groups 2 and 4 when compared to the control (*p* = 0.0154 and 0.0467, respectively), and lower urea nitrogen for Groups 3 and 4 when compared to the control (*p* = 0.0245 and 0.0570, respectively) were considered incidental and were characterized by three or more of the following: small magnitude, absence of dose dependency, inconsistency between sexes, similarity to differences present before initiation of dosing and absence of correlative findings.

**Table 4:** Summary of hematology data; mean (SD), *N* = 8

Variable (reference range and units) ^a^	Day	Group 1		Group 2		Group 3		Group 4		*P* value	
										Treatment	Time
Red blood cell count (5.8–7.8 10^6^/μL)	−17	6.23	(1.14)	5.85	(0.38)	5.75	(0.37)	5.84	(0.28)	0.3548	<0.0001
	−11	5.66	(0.34)	5.77	(0.19)	5.75	(0.27)	5.87	(0.23)		
	21	5.85	(0.49)	5.63	(0.46)	5.71	(0.48)	5.84	(0.46)		
	55	6.21	(0.59)	5.99	(0.36)	6.05	(0.42)	6.36	(0.38)		
	89	6.38	(0.51)	6.22	(0.47)	6.27	(0.26)	6.37	(0.48)		
Hemoglobin (14.1–18.7 g/dL)	−17	13.16	(2.23)	12.44	(0.72)	12.43	(0.77)	12.71	(0.56)	0.2896	<0.0001
	−11	12.05	(0.32)	12.31	(0.49)	12.55	(0.48)	12.79	(0.43)		
	21	12.49	(0.82)	12.18	(0.92)	12.50	(0.93)	12.88	(0.87)		
	55	13.68	(1.11)	13.33	(0.96)	13.70	(0.82)	14.51	(0.56)		
	89	13.88	(0.76)	13.50	(1.06)	14.13	(0.37)	14.16	(0.71)		
Hematocrit (41.0–55.3%)	−17	42.60	(7.67)	39.50	(2.38)	39.66	(2.68)	40.48	(1.89)	0.1645	<0.0001
	−11	38.48	(1.35)	38.93	(1.41)	39.68	(1.67)	40.64	(1.17)		
	21	39.18	(2.57)	37.68	(2.71)	38.98	(2.97)	40.18	(2.71)		
	55	42.58	(3.30)	41.06	(2.56)	42.58	(2.45)	44.84	(1.78)		
	89	43.29	(2.56)	41.90	(3.21)	43.86	(0.98)	44.24	(2.44)		
Platelet count (161.6–379.0 10^3^/μL)	−17	297.13	(75.13)	268.00	(106.63)	384.75	(61.17)	318.63	(64.39)	0.9909	<0.0001
	−11	320.75	(63.09)	287.75	(54.84)	366.38	(50.36)	296.75	(71.34)		
	21	323.50	(57.94)	317.88	(63.30)	352.75	(42.68)	309.00	(93.97)		
	55	300.13	(43.28)	275.00	(47.67)	326.75	(34.01)	287.75	(56.39)		
	89	275.88	(36.74)	257.50	(31.27)	308.00	(31.60)	267.38	(66.42)		
White blood cell count (7.4–14.5 10^3^/μL)	−17	9.64	(1.54)	8.27	(1.18)	9.93	(1.96)	9.34	(1.56)	0.0433	<0.0001
	−11	9.45	(1.67)	8.58	(2.08)	10.05	(2.91)	9.12	(1.53)		
	21	8.39	(1.40)	6.93	(0.70)	8.54	(2.15)	6.95	(0.91)		
	55	9.14	(2.13)	7.11	(0.98)	8.63	(2.10)	7.26	(0.51)		
	89	9.47	(1.01)	7.95	(1.20)	9.83	(2.20)	9.12	(1.62)		
Activated partial thromboplastin time (sec) ^b^	−17	11.35	(0.67)	11.56	(0.83)	11.63	(0.71)	11.44	(0.75)	0.8555	0.0859
	−11	13.68	(1.36)	13.60	(1.71)	13.05	(1.20)	13.55	(0.95)		
	21	12.74	(0.78)	12.24	(1.01)	12.24	(0.35)	12.49	(1.22)		
	55	12.26	(1.05)	12.98	(2.25)	12.61	(2.20)	11.80	(1.30)		
	89	12.13	(0.91)	12.21	(1.85)	11.76	(0.57)	11.91	(0.67)		

^a^ These reference ranges have been derived from healthy young mongrel dogs (*n* = 124) and have been provided by the animal supplier. They are to be used for information only and the two values collected before the dosing period (at Day −17 and −11) should be used for interpretation in the context of this study

^b^ Not available in mongrel dogs

**Table 5:** Summary of clinical chemistry data; mean (SD), *N* = 8

Variable (reference range and units) ^a^	Day	Group 1		Group 2		Group 3		Group 4		*P* value	
										Treatment	Time
Alanine aminotransferase (23.0–64.6 U/L)	−17	20.88	(7.12)	21.88	(4.91)	18.75	(4.65)	21.75	(5.01)	0.7693	<0.0001
	−11	21.75	(4.23)	22.00	(3.78)	21.00	(3.82)	24.38	(7.11)		
	21	25.50	(6.00)	26.50	(4.28)	24.13	(4.36)	26.88	(5.36)		
	55	28.50	(6.44)	29.25	(4.95)	27.63	(5.21)	29.50	(6.09)		
	89	32.00	(7.84)	31.13	(4.67)	29.50	(4.14)	31.13	(5.84)		
Aspartate aminotransferase (28.1–59.7 U/L)	−17	29.00	(5.04)	30.25	(4.06)	28.25	(4.27)	30.63	(2.97)	0.5585	0.0002
	−11	30.88	(3.68)	30.63	(3.46)	29.88	(2.85)	32.25	(4.46)		
	21	28.50	(3.74)	29.75	(2.60)	29.63	(3.74)	30.25	(4.62)		
	55	28.75	(3.24)	31.38	(4.50)	30.38	(6.23)	32.00	(5.50)		
	89	31.50	(5.86)	33.00	(4.07)	32.88	(3.44)	34.38	(5.04)		
Alkaline phosphatase (65.4–177.5 U/L)	−17	118.88	(22.90)	109.00	(30.81)	139.13	(32.89)	117.25	(23.92)	0.8396	<0.0001
	−11	126.88	(27.03)	114.88	(32.42)	146.63	(33.43)	120.00	(25.60)		
	21	92.88	(19.98)	86.00	(25.47)	104.50	(29.08)	88.50	(22.77)		
	55	80.13	(15.63)	75.13	(17.31)	89.13	(25.05)	76.25	(19.77)		
	89	62.75	(13.47)	56.38	(13.41)	73.63	(22.92)	59.13	(15.82)		
Urea nitrogen (10.0–22.0 mg/dL)	−17	10.25	(2.25)	10.75	(1.28)	9.75	(1.49)	10.38	(1.60)	0.0196	0.0823
	−11	12.00	(1.93)	11.50	(1.69)	11.00	(1.31)	12.38	(2.07)		
	21	16.00	(1.77)	15.50	(1.07)	13.13	(2.10)	14.50	(2.20)		
	55	16.00	(2.56)	17.13	(1.96)	14.75	(2.25)	14.88	(2.10)		
	89	16.63	(2.62)	16.38	(2.39)	14.25	(2.25)	14.63	(1.69)		
Creatinine (0.7–1.1 mg/dL)	−17	0.61	(0.06)	0.56	(0.05)	0.60	(0.08)	0.60	(0.05)	0.9290	<0.0001
	−11	0.60	(0.08)	0.59	(0.04)	0.59	(0.06)	0.59	(0.06)		
	21	0.79	(0.06)	0.78	(0.05)	0.78	(0.07)	0.81	(0.04)		
	55	0.95	(0.09)	0.94	(0.07)	0.90	(0.09)	0.93	(0.09)		
	89	1.00	(0.05)	1.00	(0.09)	0.99	(0.08)	0.98	(0.07)		
Total protein (5.3–6.4 g/dL)	−17	4.75	(0.15)	4.71	(0.14)	4.84	(0.32)	4.91	(0.20)	0.4983	<0.0001
	−11	4.71	(0.15)	4.74	(0.18)	4.85	(0.24)	4.80	(0.14)		
	21	4.98	(0.07)	4.99	(0.16)	5.09	(0.28)	5.11	(0.15)		
	55	5.23	(0.13)	5.24	(0.09)	5.35	(0.19)	5.36	(0.23)		
	89	5.10	(0.15)	5.25	(0.16)	5.36	(0.20)	5.24	(0.32)		
Albumin (2.9–3.9 g/dL)	−17	3.26	(0.15)	3.23	(0.10)	3.29	(0.19)	3.36	(0.18)	0.9691	0.1652
	−11	3.25	(0.14)	3.20	(0.09)	3.30	(0.19)	3.29	(0.12)		
	21	3.50	(0.09)	3.44	(0.13)	3.55	(0.12)	3.54	(0.15)		
	55	3.51	(0.15)	3.51	(0.19)	3.48	(0.16)	3.55	(0.15)		
	89	3.53	(0.10)	3.54	(0.13)	3.64	(0.14)	3.53	(0.20)		
Creatine kinase (127.5–869.4 U/L)	−17	365.38	(57.30)	359.38	(80.96)	342.75	(22.22)	417.88	(96.99)	0.6395	0.2253
	−11	318.38	(50.07)	322.88	(57.31)	324.75	(38.87)	403.50	(91.48)		
	21	240.88	(46.46)	289.75	(66.14)	277.88	(60.09)	336.25	(170.44)		
	55	223.25	(60.46)	248.13	(57.50)	254.00	(48.67)	302.38	(96.44)		
	89	270.00	(148.55)	255.50	(65.44)	314.38	(134.62)	285.88	(52.55)		

^a^ These reference ranges have been derived from healthy young mongrel dogs (n = 124) and have been provided by the animal supplier. They are to be used for information only and the two values collected before the dosing period (at Day −17 and −11) should be used for interpretation in the context of this study

Several animals exhibited transient clinical pathology changes (i.e. increased fibrinogen concentration, increased white blood cell count, absolute neutrophil count, absolute monocyte count) consistent with inflammation at the injection sites.

### Organ weights, gross and microscopic examinations

Summary data for organ weights are presented in Table 6. For prostate, a significant increase was only noted for Group 2 when compared to the control (*p* = 0.0364). In the absence of a dose relation and the lack of macroscopic or microscopic correlates, the observation was neither considered treatment related nor relevant.

**Table 6:** Summary of organ (g) weights; mean (SD), *N* = 8 unless stated

Organ	Group 1		Group 2		Group 3		Group 4		*P* value Treatment
Heart	170.66	(20.62)	173.22	(25.40)	174.79	(17.99)	157.73	(24.55)	0.2398
Kidneys	77.27	(9.74)	90.93	(23.45)	85.75	(17.00)	79.50	(15.42)	0.1829
Liver/Gall bladder	527.00	(73.45)	534.15	(114.49)	549.49	(129.55)	532.26	(117.27)	0.9546
Lung	180.73	(21.52)	182.79	(23.98)	180.34	(20.64)	179.20	(26.20)	0.9707
Brain	84.18	(8.19)	81.16	(7.60)	83.58	(7.48)	83.25	(7.34)	0.6928
Thymus	27.45	(12.91)	25.51	(7.78)	26.01	(6.88)	28.21	(11.79)	0.9293
Spleen	85.57	(38.68)	85.69	(34.93)	69.05	(14.38)	84.36	(37.65)	0.7196
Thyroid/Parathyroid	1.72	(0.41)	1.83	(0.43)	1.67	(0.45)	2.15	(0.84)	0.3149
Adrenals (*N* = 7^a^)	1.42	(0.24)	1.61	(0.19)	1.67	(0.27)	1.64	(0.17)	0.2064
Pituitary	0.11	(0.02)	0.10	(0.02)	0.11	(0.02)	0.09	(0.02)	0.3402
Ovary (*N* = 4)	1.39	(0.56)	1.26	(0.14)	1.17	(0.42)	1.30	(0.19)	0.8505
Uterus (*N* = 4)	6.35	(5.83)	3.04	(1.23)	3.19	(1.15)	2.88	(1.04)	0.3747
Testis (*N* = 4)	28.72	(6.37)	30.23	(4.96)	27.96	(0.90)	24.92	(6.97)	0.5779
Prostate (*N *= 4)	4.96	(2.03)	9.52	(4.75)	4.88	(1.30)	3.51	(0.87)	0.0462

^a^ Adrenal was missing in one dog

Macroscopic observations consisted of red discoloration within multiple sections of the gastrointestinal tract in two dogs in Group 2 and two dogs in Group 3, with no corresponding histopathology findings. Microscopic findings included a jejunal ulcer with minimal inflammation in one Group 2 dog, minimal cecal hemorrhage with microscopic cecal inflammation in one Group 3 dog, slight duodenal congestion in one Group 3 dog and cecal inflammation in one Group 4 dog.

Treated male dogs exhibited an increased number and severity of lymphocyte depletion within the thymus compared to the controls. The lymphocyte depletion may have been a secondary, stress-related response to the injection site findings and not a direct effect of the test article.

### Pharmacokinetic and pharmacodynamic evaluation

Predose samples on Day 1 and 69 did not contain robenacoxib nor would they be expected to since Day 1 was the first dose and Day 69 followed a 14-day washout period. Predose samples on Day 14 and 82 followed subcutaneous dosing and had low but measureable concentrations of robenacoxib in 26 of the 48 samples obtained (< 40 ng/mL).

The robenacoxib concentrations observed throughout the study at 1 and 5 h are displayed in Table 7. Increased dose resulted in increased exposure as demonstrated by 1 h concentrations and AUC_1-5h_. In view of the sparse data, there was no evidence for differences in dose-normalized exposure between the different tablet dose regimens or between tablet and subcutaneous administrations. Accumulation assessed by comparing concentrations achieved 5 h post oral administration (at Day 1 and 6 and Day 69 and 74), was not observed.

**Table 7:** Summary of robenacoxib concentration (ng/mL); mean (SD), *N *= 8

Day	Time (h)	Group 2		Group 3		Group 4	
1	1	2514	(1339)	1760	(1401)	2761	(1728)
1	5	44	(32)	151	(223)	136	(87)
6	1	1447	(1473)	1608	(1164)	2610	(2083)
6	5	30	(15)	72	(74)	73	(37)
8	1	1039	(1026)	1677	(1477)	4991	(4129)
8	5	78	(80)	212	(149)	191	(154)
11	1	1564	(582)	2014	(573)	3015	(1336)
11	5	435	(202)	1126	(344)	1913	(1101)
14	1	2825	(3489)	1603	(1442)	1822	(1413)
14	5	38	(43)	110	(118)	103	(63)
69	1	2896	(1369)	2653	(2530)	3915	(2533)
69	5	53	(54)	128	(105)	154	(129)
74	1	1290	(955)	1232	(1632)	4759	(2889)
74	5	67	(52)	157	(180)	104	(61)
76	1	3467	(2004)	2791	(2447)	4492	(4924)
76	5	166	(178)	366	(276)	1047	(1421)
79	1	1269	(405)	1402	(386)	2245	(1137)
79	5	944	(299)	2050	(428)	3133	(999)
82	1	1130	(563)	2118	(1349)	2541	(2049)
82	5	45	(50)	110	(113)	114	(126)

The predicted COX-1 and COX-2 inhibition levels corresponding to the robenacoxib concentrations observed at 1 and 5 h throughout the study were calculated (Table 8). At the dosages tested in this study and 1 h after the treatment, the mean inhibition was 23–29% for COX-1 and 79–87% for COX-2 but there were no significant differences among the dose groups for the inhibition levels (*p* = 0.2694 and 0.7723 for COX-1 and COX-2 inhibition, respectively). At 5 h after the treatment, inhibition decreased and the mean inhibition was 6–11% for COX-1 and 24–41% for COX-2 (Table 8). The difference between the groups was greater at 5 h and the level of inhibition for both COX-1 and COX-2 was statistically significantly higher with increasing doses (*p* < 0.0001).

**Table 8:** Predicted inhibition percentage of COX-1 and COX-2 for the observed robenacoxib concentrations at 1 and 5 h; mean (SD) [max], *N* = 80

	Time	Group 2			Group 3			Group 4		
% inhibition COX-1	1	24	(9)	[50]	23	(10)	[44]	29	(11)	[53]
% inhibition COX-1	5	6	(5)	[22]	10	(8)	[30]	11	(10)	[36]
% inhibition COX-2	1	82	(23)	[99]	79	(27)	[98]	87	(20)	[99]
% inhibition COX-2	5	24	(27)	[88]	39	(32)	[95]	41	(32)	[97]

## Discussion

The primary objective of this study was to evaluate the safety of the interchangeable use of Onsior™ tablets and Onsior™ solution for injection containing robenacoxib in dogs. The dose levels and dose multiples (up to 3X) were chosen in accordance with Target Animal Safety Guidelines for Veterinary Pharmaceutical Products (VICH GL43 [16]) to identify a margin of safety. This modification from the traditional approach of using 1X, 3X and 5X dose levels in target animal safety studies was based on animal welfare concerns and preventing generation of data already available as well as assessing safety in young dogs.

Alternating regimens of robenacoxib tablets and injection (Onsior™) for three 20 day cycles (each separated by a 14-day washout) for 88 days to 4 month old dogs was well tolerated based on the overall good health of the dogs. Primary treatment-related abnormalities were confined to (i) microscopic findings in the gastrointestinal tract and (ii) macroscopic and microscopic findings at the subcutaneous injection sites.

The principal targets for toxicity of non-selective NSAIDs are the gastrointestinal tract, kidney, liver and inhibition of blood clotting [25, 26]. Robenacoxib-related gastrointestinal microscopic findings seen in this study were consistent with the known pharmacology of NSAID class of COX-2 inhibitors [27] and with previous studies evaluating the safety/toxicity of robenacoxib administered separately orally or via subcutaneous injection [4, 6]. They were mild and did not result in clinical signs, changes in body weight, or effects on haematology. Microscopic findings were generally higher in incidence and severity among dogs in Groups 3 and 4 compared to those in Group 2. In addition, there were no changes in serum total protein or albumin concentrations which could indicate protein-losing enteropathy.

There was no evidence from this study of toxicity of robenacoxib on the kidney. There were no post mortem gross changes and no relevant histological findings affecting the kidney. Plasma creatinine, urea concentrations and results of urinalysis were normal in the treated groups. There were no post mortem gross changes and no relevant histological findings affecting the liver. Analysis of alkaline phosphatase, alanine aminotransferase and aspartate aminotransferase did not indicate any effect on the liver function. Finally, there was no effect of robenacoxib on the coagulation parameters (i.e., activated partial thromboplastin time, BMBT) similar to what has been previously reported for robenacoxib in both cats and dogs [6, 11].

The clinical signs observed upon subcutaneous administration (twitching, scratching, licking or vocalization) were uncommon and, as they occasionally occur after subcutaneous injection in dogs, were not considered related to robenacoxib injectable or saline, but to the subcutaneous injection process itself.

The incidence of macroscopic findings at the subcutaneous dose sites was greater in Groups 3 or 4 than in Groups 1 or 2. Following injection, severity was generally dose-related and the findings at the subcutaneous injection sites were consistent with the observations in a previous target animal safety study using only Onsior™ solution for injection (unpublished report). In this study, 6-month old healthy beagles were administered subcutaneously Onsior™ solution for injection formulation at 0 (control), 2, 6 and 20 mg/kg of robenacoxib during three dosing cycles of one subcutaneous injection per day for three consecutive days separated by 11-day washout time. A dose-dependent increased frequency of thickening scabbing, scarring, discoloration foci, accentuated vasculature, and subcutaneous haemorrhage at the injection site, were observed at necropsy. Subcutaneous inflammation was dose-dependent and a progressive resolution of subcutaneous lesions over time was evident. Thus, it can be concluded that the interchangeable use with Onsior™ tablets does not result in additional effects compared to use of Onsior™ solution for injection only.

In the present study, none of these findings had a clinically significant effect on the health or well-being of the animals. Dogs were comfortable and healthy, bright, alert, and responsive, hydrated, and in good body condition, showing only some discomfort upon palpation of dose sites.

The findings of lipid infiltrates and infiltrates of vacuolated macrophages in axillary lymph nodes were considered secondary treatment-related effects because they represented drainage of the necrosis and degeneration that occurred at the subcutaneous injection sites (including a control dog). Likewise, depletion of lymphocytes in the thymus may have been a secondary response and not a direct effect of the treatment.

Toxicokinetic results confirmed consistent and appropriate exposure of the animals and thus allows conclusion to be made regarding the safety of interchangeable use. Further interpretation of the toxicokinetics in this study is made difficult by the limited sampling schedule. A maximum of three (one predose and two post dose) samples during a dosing interval is insufficient to accurately estimate C_max_, half-life, or AUC. No accumulation was observed in this 88-day study. Previous studies have shown robenacoxib to be eliminated in the dog with a half-life of less than 1 h [21] therefore no accumulation would be expected with once daily dosing.

The predicted levels of COX-1 and COX-2 inhibition in this study were derived from the concentrations observed at 1 and 5 h. In general, the maximum inhibition of COX-1 and COX-2 was observed at 1 h where high average inhibition of COX-2 (i.e., ≥ 79%) and limited inhibition of COX-1 (≤ 41%) was predicted. The maximum predicted inhibition for COX-1 was 53%, in Group 4 (Table 8). Inhibition levels were higher when the blood concentrations were higher at 1 h after treatment, but without marked differences between the dose groups presumably because the concentrations achieved in all groups had saturated the enzyme and reflect maximum activity. Levels of COX-1 and COX-2 inhibition were decreased at 5 h compared to at 1 h corresponding to decreased blood concentrations. At these lower concentrations, the enzymes are not saturated and therefore at 5 h the level of inhibition was dose-dependent. The magnitude and duration of COX-1 inhibition needed to induce gastrointestinal toxicity is not known, however it was concluded in a previous study that approximately 50% inhibition of COX-1 for up to 3.5 h or 25% for up to 8 h was safe for robenacoxib and the canine gastrointestinal tract [6]. This is in alignment with the maximum inhibition of COX-1 predicted in the present study, in which gastrointestinal toxicity was not observed.

The results of this study indicate that injections of robenacoxib can be safely administered instead of a tablet to a dog receiving oral therapy or that robenacoxib injection(s) can be followed by oral robenacoxib therapy. The microscopic findings in the gastrointestinal tract and the clinical signs and microscopic findings at the injection site were as expected based on a previous study with robenacoxib solution for injection alone (unpublished report) and the known properties of this class of compound and mode of administration. There were no safety concerns caused by the interchangeable use of the oral and injectable robenacoxib formulations. To our knowledge, this is the first publication showing results of a study designed to establish the interchangeable use of two formulations in veterinary medicine.

## Conclusions

This study supports the safe use of any combination of Onsior™ tablets or injectable once daily. There were no serious adverse events during the course of the study. No changes in body weight, food consumption, ophthalmic examinations, electrocardiograms, buccal mucosal blood times, clinical pathology or organ weight were attributable to robenacoxib administration. Primary treatment-related findings were confined to clinical signs upon subcutaneous administration, macroscopic and microscopic findings at the subcutaneous injection sites and microscopic findings in the gastrointestinal tract, typically seen with the individual use of oral or injectable robenacoxib or with this class of compound and mode of administration. It is concluded that alternating regimens of robenacoxib tablets and injection (Onsior™) were well tolerated in healthy young dogs when administered once a day.

## Abbreviations

AUC: Area under the curve
BMBT: Buccal mucosal bleed times
C_max_: Maximum blood drug concentration
COX: Cyclooxygenase
ECG: Electrocardiogram
FDA-CVM: Food and Drug Administration’s Center for Veterinary Medicine
HPLC: high-performance liquid chromatography
K2-EDTA: Ethylenediaminetetraacetic acid dipotassium
LC-MS/MS: Liquid chromatography tandem mass spectrometry
LLOQ: Lower limit of quantification
NSAID: Non-steroidal anti-inflammatory drug
PGE_2_: Prostaglandin E2
SC: Subcutaneous
T_max_: Time to maximum blood concentration following drug administration
TxB_2_: Thromboxane B2
VICH: Veterinary International Conference on Harmonization
